# (*Z*)-5-(4-Fluoro­benzyl­idene)-1,3-thia­zolidine-2,4-dione

**DOI:** 10.1107/S1600536807068316

**Published:** 2008-01-25

**Authors:** Hong-Shun Sun, Ye-Ming Xu, Wei He, Shi-Gui Tang, Cheng Guo

**Affiliations:** a Department of Applied Chemistry, College of Sciences, Nanjing University of Technolgy, Xinmofan Road No. 5, Nanjing 210009, People’s Republic of China

## Abstract

In the title compound, C_10_H_6_FNO_2_S, the benzene and thia­zolidine rings make a dihedral angle of 7.52 (3)°. Intra­molecular C—H⋯O and C—H⋯S hydrogen bonds result in the formation of nearly planar five- and six-membered rings; the adjacent rings are nearly coplanar. In the crystal structure, inter­molecular N—H⋯O hydrogen bonds link the mol­ecules.

## Related literature

For general background, see: Barreca *et al.* (2002 ▶); Botti *et al.* (1996 ▶). For a related structure, see: Guo *et al.* (2006 ▶). For bond-length data, see: Allen *et al.* (1987 ▶).
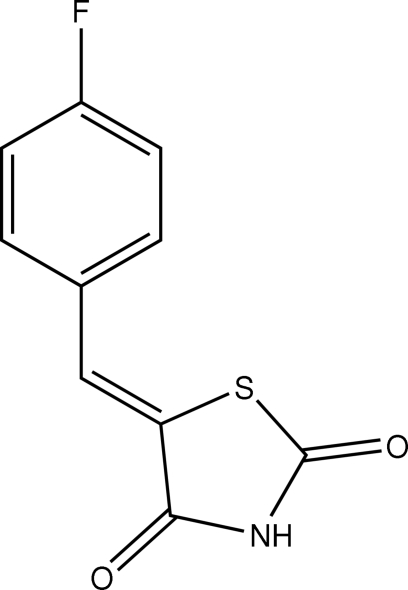


## Experimental

### 

#### Crystal data

C_10_H_6_FNO_2_S
*M* *_r_* = 223.22
Orthorhombic, 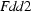

*a* = 26.519 (5) Å
*b* = 36.509 (7) Å
*c* = 3.8490 (8) Å
*V* = 3726.6 (13) Å^3^
*Z* = 16
Mo *K*α radiation
μ = 0.34 mm^−1^
*T* = 294 (2) K
0.30 × 0.10 × 0.10 mm

#### Data collection

Enraf–Nonius CAD-4 diffractometer
Absorption correction: ψ scan (North *et al.*, 1968 ▶) *T*_min_ = 0.905, *T*_max_ = 0.967
2087 measured reflections
1059 independent reflections
790 reflections with *I* > 2σ(*I*)
*R*_int_ = 0.042
3 standard reflections frequency: 120 min intensity decay: none

#### Refinement

*R*[*F* ^2^ > 2σ(*F* ^2^)] = 0.053
*wR*(*F* ^2^) = 0.121
*S* = 1.04
1059 reflections
136 parameters
1 restraint
H-atom parameters constrained
Δρ_max_ = 0.38 e Å^−3^
Δρ_min_ = −0.66 e Å^−3^
Absolute structure: Flack (1983 ▶), no Friedel pairs
Flack parameter: 0.0 (2)

### 

Data collection: *CAD-4 Software* (Enraf–Nonius, 1989 ▶); cell refinement: *CAD-4 Software*; data reduction: *XCAD4* (Harms & Wocadlo, 1995 ▶); program(s) used to solve structure: *SHELXS97* (Sheldrick, 2008 ▶); program(s) used to refine structure: *SHELXL97* (Sheldrick, 2008 ▶); molecular graphics: *SHELXTL* (Siemens, 1996 ▶); software used to prepare material for publication: *SHELXL97*.

## Supplementary Material

Crystal structure: contains datablocks global, I. DOI: 10.1107/S1600536807068316/hk2407sup1.cif

Structure factors: contains datablocks I. DOI: 10.1107/S1600536807068316/hk2407Isup2.hkl

Additional supplementary materials:  crystallographic information; 3D view; checkCIF report

## Figures and Tables

**Table 1:** Hydrogen-bond geometry (Å, °)

*D*—H⋯*A*	*D*—H	H⋯*A*	*D*⋯*A*	*D*—H⋯*A*
N—H0*A*⋯O2^i^	0.86	1.98	2.830 (5)	171
C5—H5*A*⋯S	0.93	2.54	3.241 (5)	133
C7—H7*A*⋯O2	0.93	2.50	2.870 (5)	104

Symmetry code: (i) 
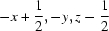
.

**Fig. 1.**
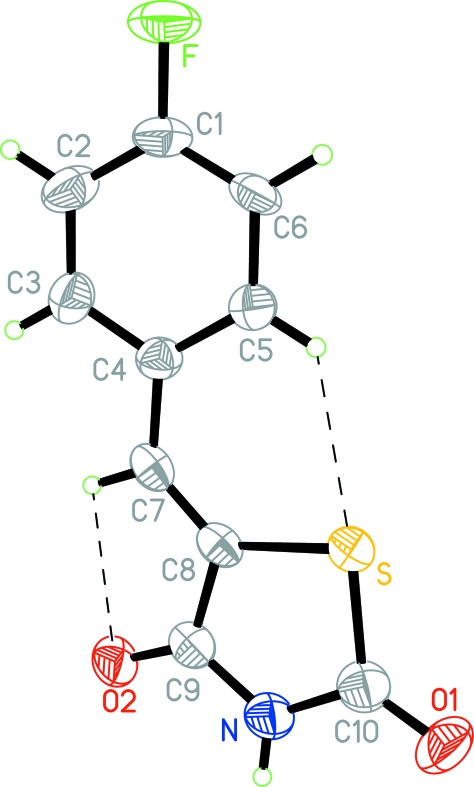
The molecular structure of the title molecule, with the atom-numbering scheme. Displacement ellipsoids are drawn at the 30% probability level. Hydrogen bonds are shown as dashed lines.

## supplementary crystallographic information

### Comment

Thiazolidines are an important class of heteroaromatic compounds and have widespread applications from pharmaceuticals (Barreca *et al.*, 2002) to materials (Botti *et al.*, 1996). As part of our ongoing studies in this area (Guo *et al.*, 2006), we report herein the synthesis and crystal structure of the title compound, (I).

In the molecule of (I) (Fig. 1), bond lengths and angles are within normal ranges (Allen *et al.*, 1987). The intramolecular C—H···O and C—H···S hydrogen bonds (Table 1) result in the formations of nearly planar five- and six-membered rings; C (S/H5A/C4/C5/C7/C8) and D (O2/H7A/C7—C9). Rings A (C1—C6) and B (N/S/C8—C10) are, of course, planar and the dihedral angles between them are A/B = 7.52 (3)°, A/C = 4.73 (3)°, A/D = 6.90 (3)°, B/C = 3.63 (2)°, B/D = 2.39 (3)° and C/D = 4.56 (2)°. So, the adjacent rings are also nearly co-planar.

In the crystal structure, intermolecular N—H···O hydrogen bonds (Table 1) link the molecules, in which they may be effective in the stabilization of the structure.

### Experimental

For the preparation of the title compound, thiazolidine-2,4-dione (10 mmol) and 4-fluorobenzaldehyde (10 mmol) were dissolved in ethanol (10 ml) in a round-bottomed flask (50 ml) and 5 drops of piperidine were added. The flask was heated in a modified domestic microwave oven at 300 W for 5 min. After cooling, the mixture was poured into water and the crude compound (I) filtered out. Crystals of (I) suitable for X-ray analysis were obtained by slow evaporation of an ethanol solution.

### Refinement

H atoms were positioned geometrically, with N—H = 0.86 Å (for NH) and C—H = 0.93 Å for aromatic H, and constrained to ride on their parent atoms, with *U*_iso_(H) = 1.2*U*_eq_(C,*N*).

### Crystal data

**Table:**

C_10_H_6_FNO_2_S	*F*_000_ = 1824
*M**_r_* = 223.22	*D*_x_ = 1.591 Mg m^−^^3^
Orthorhombic, *F**d**d*2	Mo *K*α radiation λ = 0.71073 Å
Hall symbol: F 2 -2d	Cell parameters from 25 reflections
*a* = 26.519 (5) Å	θ = 9–13º
*b* = 36.509 (7) Å	µ = 0.34 mm^−^^1^
*c* = 3.8490 (8) Å	*T* = 294 (2) K
*V* = 3726.6 (13) Å^3^	Block, colorles
*Z* = 16	0.30 × 0.10 × 0.10 mm

### Data collection

**Table:**

Enraf–Nonius CAD-4 diffractometer	*R*_int_ = 0.042
Radiation source: fine-focus sealed tube	θ_max_ = 26.0º
Monochromator: graphite	θ_min_ = 1.9º
*T* = 294(2) K	*h* = 0→32
ω/2θ scans	*k* = 0→44
Absorption correction: ψ scan(North *et al.*, 1968)	*l* = −4→0
*T*_min_ = 0.905, *T*_max_ = 0.967	3 standard reflections
2087 measured reflections	every 120 min
1059 independent reflections	intensity decay: none
790 reflections with *I* > 2σ(*I*)	

### Refinement

**Table:**

Refinement on *F*^2^	Hydrogen site location: inferred from neighbouring sites
Least-squares matrix: full	H-atom parameters constrained
*R*[*F*^2^ > 2σ(*F*^2^)] = 0.053	*w* = 1/[σ^2^(*F*_o_^2^) + (0.05*P*)^2^ + 3*P*] where *P* = (*F*_o_^2^ + 2*F*_c_^2^)/3
*wR*(*F*^2^) = 0.121	(Δ/σ)_max_ < 0.001
*S* = 1.04	Δρ_max_ = 0.38 e Å^−^^3^
1059 reflections	Δρ_min_ = −0.66 e Å^−^^3^
136 parameters	Extinction correction: none
1 restraint	Absolute structure: Flack (1983), no Friedel pairs
Primary atom site location: structure-invariant direct methods	Flack parameter: 0.0 (2)
Secondary atom site location: difference Fourier map	

### Special details

**Table:**

Geometry. All e.s.d.'s (except the e.s.d. in the dihedral angle between two l.s. planes) are estimated using the full covariance matrix. The cell e.s.d.'s are taken into account individually in the estimation of e.s.d.'s in distances, angles and torsion angles; correlations between e.s.d.'s in cell parameters are only used when they are defined by crystal symmetry. An approximate (isotropic) treatment of cell e.s.d.'s is used for estimating e.s.d.'s involving l.s. planes.
Refinement. Refinement of *F*^2^ against ALL reflections. The weighted *R*-factor *wR* and goodness of fit *S* are based on *F*^2^, conventional *R*-factors *R* are based on *F*, with *F* set to zero for negative *F*^2^. The threshold expression of *F*^2^ > σ(*F*^2^) is used only for calculating *R*-factors(gt) *etc*. and is not relevant to the choice of reflections for refinement. *R*-factors based on *F*^2^ are statistically about twice as large as those based on *F*, and *R*- factors based on ALL data will be even larger.

### Fractional atomic coordinates and isotropic or equivalent isotropic displacement parameters (Å^2^)

**Table:**

	*x*	*y*	*z*	*U*_iso_*/*U*_eq_	
S	0.08943 (4)	−0.00189 (3)	−0.0089 (5)	0.0379 (3)	
F	−0.06109 (11)	0.13395 (8)	0.6207 (12)	0.0681 (12)	
O1	0.13745 (13)	−0.05739 (9)	−0.3062 (14)	0.0619 (12)	
O2	0.21942 (10)	0.03754 (8)	0.2142 (14)	0.0493 (10)	
N	0.18501 (14)	−0.01228 (9)	−0.0627 (14)	0.0425 (11)	
H0A	0.2136	−0.0219	−0.1150	0.051*	
C1	−0.01787 (17)	0.11646 (12)	0.5430 (15)	0.0416 (12)	
C2	0.02675 (18)	0.13443 (12)	0.5921 (16)	0.0484 (15)	
H2A	0.0273	0.1584	0.6744	0.058*	
C3	0.07078 (19)	0.11621 (11)	0.5166 (17)	0.0444 (13)	
H3A	0.1015	0.1278	0.5559	0.053*	
C4	0.07035 (16)	0.08082 (10)	0.3826 (13)	0.0346 (11)	
C5	0.02378 (16)	0.06382 (12)	0.3314 (15)	0.0400 (12)	
H5A	0.0225	0.0402	0.2421	0.048*	
C6	−0.02016 (15)	0.08195 (12)	0.4125 (16)	0.0452 (14)	
H6A	−0.0512	0.0707	0.3782	0.054*	
C7	0.11827 (16)	0.06366 (11)	0.3095 (14)	0.0352 (10)	
H7A	0.1462	0.0772	0.3782	0.042*	
C8	0.12929 (15)	0.03151 (11)	0.1597 (14)	0.0317 (10)	
C9	0.18224 (17)	0.02026 (11)	0.1107 (15)	0.0370 (12)	
C10	0.14053 (17)	−0.02927 (12)	−0.1510 (17)	0.0450 (13)	

### Atomic displacement parameters (Å^2^)

**Table:**

	*U*^11^	*U*^22^	*U*^33^	*U*^12^	*U*^13^	*U*^23^
S	0.0303 (5)	0.0391 (5)	0.0444 (6)	−0.0005 (4)	0.0015 (7)	−0.0028 (6)
F	0.0400 (16)	0.0705 (18)	0.094 (3)	0.0212 (14)	0.003 (2)	−0.024 (2)
O1	0.058 (2)	0.0465 (17)	0.081 (3)	0.0105 (16)	0.000 (3)	−0.026 (2)
O2	0.0267 (16)	0.0444 (16)	0.077 (3)	0.0002 (12)	−0.0039 (19)	0.001 (2)
N	0.0295 (18)	0.0403 (18)	0.058 (3)	0.0068 (15)	0.001 (2)	0.001 (2)
C1	0.033 (2)	0.051 (2)	0.041 (3)	0.012 (2)	0.003 (2)	−0.009 (3)
C2	0.047 (3)	0.042 (2)	0.056 (4)	0.012 (2)	−0.009 (3)	−0.018 (3)
C3	0.047 (3)	0.036 (2)	0.050 (3)	−0.0028 (19)	−0.002 (3)	−0.007 (3)
C4	0.031 (2)	0.036 (2)	0.036 (3)	0.0030 (17)	−0.002 (2)	−0.002 (2)
C5	0.035 (2)	0.038 (2)	0.047 (3)	0.0012 (17)	−0.001 (2)	−0.006 (2)
C6	0.0209 (18)	0.056 (3)	0.058 (4)	−0.0019 (18)	0.007 (2)	−0.018 (3)
C7	0.030 (2)	0.042 (2)	0.033 (3)	−0.0057 (17)	0.000 (2)	0.008 (2)
C8	0.024 (2)	0.040 (2)	0.031 (3)	0.0007 (16)	−0.003 (2)	0.009 (2)
C9	0.032 (2)	0.037 (2)	0.042 (3)	0.0039 (18)	−0.001 (2)	0.011 (2)
C10	0.038 (2)	0.042 (2)	0.055 (4)	0.0065 (19)	−0.002 (3)	0.002 (3)

### Geometric parameters (Å, °)

**Table:**

S—C8	1.739 (4)	C2—H2A	0.9300
S—C10	1.770 (5)	C3—C4	1.391 (6)
F—C1	1.346 (5)	C3—H3A	0.9300
O1—C10	1.191 (6)	C4—C5	1.396 (6)
O2—C9	1.237 (5)	C4—C7	1.445 (6)
N—C9	1.364 (6)	C5—C6	1.376 (6)
N—C10	1.375 (6)	C5—H5A	0.9300
N—H0A	0.8600	C6—H6A	0.9300
C1—C6	1.358 (6)	C7—C8	1.340 (6)
C1—C2	1.366 (7)	C7—H7A	0.9300
C2—C3	1.375 (6)	C8—C9	1.475 (6)
			
C8—S—C10	92.6 (2)	C6—C5—H5A	119.9
C9—N—C10	117.9 (4)	C4—C5—H5A	119.9
C9—N—H0A	121.1	C1—C6—C5	119.5 (4)
C10—N—H0A	121.1	C1—C6—H6A	120.3
F—C1—C6	119.0 (4)	C5—C6—H6A	120.3
F—C1—C2	118.6 (4)	C8—C7—C4	131.0 (4)
C6—C1—C2	122.4 (4)	C8—C7—H7A	114.5
C1—C2—C3	118.3 (4)	C4—C7—H7A	114.5
C1—C2—H2A	120.9	C7—C8—C9	120.4 (4)
C3—C2—H2A	120.9	C7—C8—S	129.9 (3)
C2—C3—C4	121.4 (4)	C9—C8—S	109.6 (3)
C2—C3—H3A	119.3	O2—C9—N	124.0 (4)
C4—C3—H3A	119.3	O2—C9—C8	125.1 (4)
C3—C4—C5	118.2 (4)	N—C9—C8	110.9 (4)
C3—C4—C7	117.9 (4)	O1—C10—N	124.9 (4)
C5—C4—C7	123.9 (4)	O1—C10—S	126.1 (4)
C6—C5—C4	120.2 (4)	N—C10—S	109.0 (3)
			
F—C1—C2—C3	179.1 (5)	C4—C7—C8—S	0.6 (9)
C6—C1—C2—C3	−2.2 (9)	C10—S—C8—C7	177.2 (5)
C1—C2—C3—C4	2.1 (9)	C10—S—C8—C9	−1.5 (4)
C2—C3—C4—C5	−1.0 (8)	C10—N—C9—O2	177.5 (5)
C2—C3—C4—C7	179.8 (5)	C10—N—C9—C8	−1.9 (6)
C3—C4—C5—C6	0.0 (8)	C7—C8—C9—O2	3.9 (8)
C7—C4—C5—C6	179.1 (5)	S—C8—C9—O2	−177.2 (4)
F—C1—C6—C5	179.8 (5)	C7—C8—C9—N	−176.7 (4)
C2—C1—C6—C5	1.2 (9)	S—C8—C9—N	2.2 (5)
C4—C5—C6—C1	−0.1 (9)	C9—N—C10—O1	178.9 (6)
C3—C4—C7—C8	−174.7 (5)	C9—N—C10—S	0.7 (6)
C5—C4—C7—C8	6.3 (9)	C8—S—C10—O1	−177.6 (6)
C4—C7—C8—C9	179.3 (5)	C8—S—C10—N	0.6 (5)

### Hydrogen-bond geometry (Å, °)

**Table:**

*D*—H···*A*	*D*—H	H···*A*	*D*···*A*	*D*—H···*A*
N—H0A···O2^i^	0.86	1.98	2.830 (5)	171
C5—H5A···S	0.93	2.54	3.241 (5)	133
C7—H7A···O2	0.93	2.50	2.870 (5)	104

Symmetry codes: (i) −*x*+1/2, −*y*, *z*−1/2.
